# Intrafasciomembranal Fluid Pressure: A Novel Approach to the Etiology of Myalgias, Part II

**DOI:** 10.7759/cureus.35163

**Published:** 2023-02-19

**Authors:** Stig Runar Hopen

**Affiliations:** 1 Research, HOPEn Muskelterapi, Beitstad, NOR

**Keywords:** weather-related pain, densification, thickening of fascia, myofascial chains, tissue length, intramuscular fluid pressure, intrafasciomembranal fluid pressure, fasciomembrane, fascia, myalgia

## Abstract

The fascia forms a hierarchy of spaces (small and large compartments) that contain and enclose muscle fibers, fiber bundles, skeletal muscles, and compartments of several skeletal muscles. Solid fascia serves as a membrane that enables an increased volume and fluid pressure within such a fasciomembrane, an intrafasciomembrial fluid pressure (IFMFP). Increased IFMFP provides a theoretical model and a common explanation for the etiology of the myalgias: trigger point (TrP), chronic exertional compartment syndrome (CECS), overtraining syndrome (OTS), and delayed onset muscle soreness (DOMS). Many myalgias and their symptoms are poorly understood, and this review aims to provide an extension to this theoretical model and novel approach. This review suggests that the swelling from elevated IFMFP also likely leads to a longitudinal shortening of the same affected tissue. This model of swelling and shortening provides additional explanations for the changes in the lines of force through the body that can lead to changes in the body's posture and, thus, to compensatory movements. This new approximation also provides a biomechanical explanation for the thickening of the fascia and referred pain, and also suggests that IFMFP is a factor in weather-related pain.

## Introduction and background

Many myalgias and their symptoms are poorly understood. In the first review article by the author, "Intrafasciomembranal Fluid Pressure: A Novel Approach to the Etiology of Myalgias" [1], the total societal costs associated with musculoskeletal disorders for Norway in 2016, were found as high as up to Norwegian krone (NOK) 255 billion. Due to the high costs, a clearer understanding of mechanistic disruption is needed. In the same article, similarities between myofascial trigger point (TrP), chronic exertional compartment syndrome (CECS), overtraining syndrome (OTS), and delayed onset muscle soreness (DOMS) were identified. The common denominators provided a valid theoretical model for the etiology of these myalgias. Further, the literature reviewed suggested that extreme exertion or static muscle loading for extended periods of time may cause myalgia due to impaired fluid flow. This first article also stated that solid fascia is a continuous membrane that forms a hierarchy of spaces (small and large compartments) that contain and enclose muscle fibers, fiber bundles, skeletal muscles, and compartments, and this fasciomembrane enables differentiation of fluid and an increased fluid volume within it. Regardless of the anatomical level, this generates an increased intrafasciomembranal fluid pressure (IFMFP)

**Figure 1:**
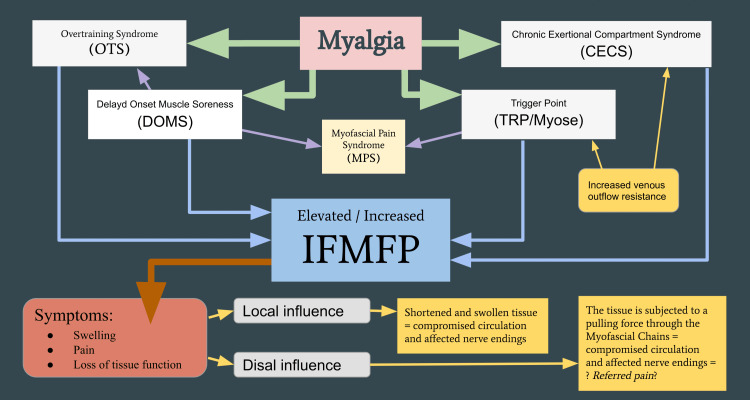
Myalgia and IFMFP symptoms and influence IFMFP: intrafasciomembranal fluid pressure Image credit: Author

This review (Part II) aims to provide a possible explanation for why swelling from elevated IFMFP can be also seen in the context of densification and thickening of the fascia, altered lines of force in the myofascia through the body (local and distal influence) (Figure 1), referred pain, as well as a part of weather-related pain.

## Review

Tissue length

**Figure 2:**
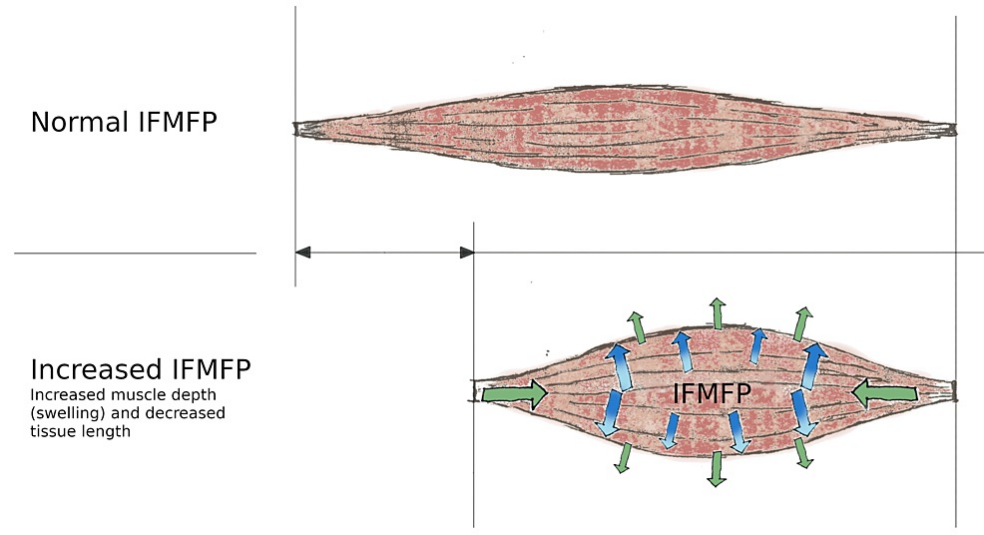
Increased pressure causes swelling that shortens tissue length at all levels (fibers, bundles, muscles, compartments) IFMFP: intrafasciomembranal fluid pressure Image credit: Author

**Figure 3:**
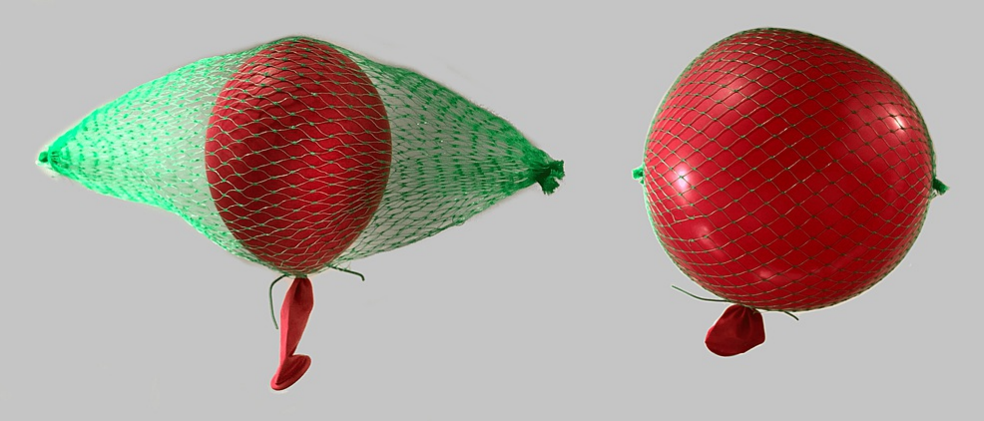
A simple balloon and net model demonstrates pressure impact; as the pressure and diameter of the balloon increase, the longitudinal direction of the net decreases and the net takes on a rounder shape Image credit: Author

Fiber architecture in muscles is not fixed but changes dynamically during contraction. Ultrasound measurements have shown that shortening also leads to muscle thickening (increased muscle depth) [2]. The redistribution of strain-energy potentials through the muscle tissue during contractions shows how fiber shortening, pennation angle, and transverse bulging in the stress and strain of the muscle tissue are related to the interaction between the material properties and the action of the contractile elements [3]. Some studies show that passively stretched muscles give longer and narrower aponeurosis, while the aponeurosis became both longer and wider with voluntary muscle activity, probably as a result of pressure from the muscle when the shape changes [4]. Increased muscle depth (swelling) from IFMFP is also likely to shorten the muscle in the longitudinal direction prior to voluntary contraction (Figure 2), as this volume change is dependent on length [5]. This can be demonstrated in a simple model where the pressure will apply in the same way as when you inflate a balloon inside a coil-shaped net, where the net represents a fasciomembrane and the pressure is expressed through the balloon; as the pressure and diameter of the balloon increase, the longitudinal direction of the net decreases and the net takes on a rounder shape(Figure 3). The principle is similar to what we find in Poisson's ratio [6].

IFMFP impact on surrounding tissues

Contractile forces act in the musculoskeletal system through myofascia to the skeleton. A systematic review suggests that most skeletal muscles in the human body are directly bound by connective tissue [7]. Myofascial chains, meridians, lines, or anatomy trains, have been identified in connecting muscles, tendons, and ligaments throughout the body [8]. Muscles should not be thought of as individual actuators, as tension in one area of the myofascial chain can be transmitted to other parts of the body by the myofascial web [7], and dysfunctions and pain can be far removed from the source through this connecting system [9].

Several models show a theoretical representation of active and passive forces in the human body through a fascial continuum. The models' biotensegrity, fascintegrity, and myofascial chains are all based on valid concepts [10]. The term biotensegrity is derived from the concept of biological tension and integrity. Transmission of mechanical tension (active or passive) determines a constant adaptation of the body structure and posture, without damaging or deforming the integrity of the shape and function. This concept can be found throughout the body and the principle can be transferred to contracting muscles as well as all the way down to a single cell [11]. Fascintegrity additionally includes tension caused by the nerve tissue, the vascular tissue, and the movement of body fluids (liquid fascia) such as blood, lymph, and interstitial and intracellular fluid [11]. Myofascial chains reflect the fascial continuum, Myers laid the foundation for myofascial chains [8], while Stecco in 1988 saw muscle continuity and acupuncture meridians [12]. The concept explains that the tension in a contractile area in a myofascial chain has consequences and affects other areas both near and far. From a microscopic and macroscopic point of view, it has been shown through animal, cadaver, and in vivo studies that myofascial tissue can transfer tension to other muscles [12,13]. Biotensegrity, fascintegrity, and myofascial chains do not exclude but rather complement each other in such a way that it is natural to think that biotensegrity and fascintegrity are both important factors in myofascial chains and vice versa.

Thickening and altered lines of force

Thickening of the fascia is the tissue's natural adaptation to the increased load from muscle use over a period of time. When the tissue repeatedly is subjected to challenging stress stimuli, fibroblasts continuously but slowly adapt the morphology [14]. It is also believed that changes in the concentration and molecular structure of hyaluronic acid (HA) result in a limitation of gliding called densification [15]. Research has revealed an association between increased fascia thickness and reduced joint flexibility in patients with chronic pain [16]. Mechanical load from increased IFMFP will both locally and in the associated tissue likely lead to an adaptation and an alteration (thickening) of the fascia and change the sliding properties of the fascia (densification) providing replacement lines of force transmission within the myofascia, as a response to the increased load [17]. Through biotensegrity, fascintegrity, and myofascial chains in the fascial continuum, this strain and altered lines of force derived from increased IFMFP will affect the body and the body's posture, potentially leading to compensatory movements.

Referred pain

**Figure 4:**
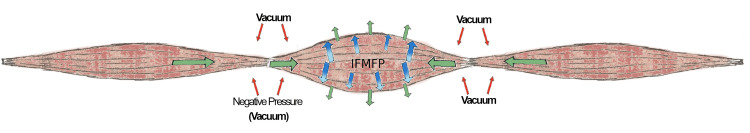
Impact of increased IFMFP on a myofascial chain IFMFP: intrafasciomembranal fluid pressure Image credit: Author

Load transfer along meridians opens a new frontier for understanding referred pain [7]. An elevated IFMFP causes the tissue to change its shape. The elevated pressure increases the depth of the affected tissue, and this will squeeze the lateral tissue. The increasing depth leads to the shortening of the same IFMFP-affected tissue and also creates a longitudinal mechanical traction force on neighboring joints and muscles through the myofascial chain (Figure 4). The mechanics are similar to those under a normal voluntary contraction. The pulling load transfer of the adjacent tissue in the longitudinal direction makes this neighboring tissue longer and, at the same time, lose depth. This narrowed tissue will also provide a secondary squeeze on blood and lymph capillaries and nerve endings, and thus predispose to reduced metabolism, ischemia, densification, and inflicted mechanical pain in this adjacent tissue also and will also affect the pressure outside the tendons or other stretched tissue. As a result of this reduced depth, this will give a negative pressure (vacuum) in the surrounding tissue of the tendon [18].

An example of the mechanism in referred pain might be a TrP, described as a tight band containing a hyperirritable palpable point [19]. The hyperirritable point will be a focal area consisting of muscle fibers with increased IFMFP and the described tight band will be the tissue of the referred pain in the longitudinal direction, which is subjected to a pulling force from the shortening in the hyperirritable point.

All of these pushing and pulling forces are likely key factors in the phenomenon of referred pain, and this also provides a possible explanation for the "knots" and "threads", observed with palpation.

Weather-related pain

Another phenomenon that theoretically can be linked to IFMFP is weather-related pain. It is a common belief is that the weather affects pain. Pressure pain tolerance and cold pain tolerance correlate with meteorological variables, and these findings suggest that weather has a causal and dynamic effect on pain tolerance [20]. Alterations in barometric pressure (BMP) have also been associated with triggering tension-type headaches but multiple studies have been performed with inconsistent results [21]. Fagerlund et al. found that pain levels in fibromyalgia patients can change according to weather conditions [22]. In their study, low BMP and increased humidity were significantly associated with increased pain intensity and pain discomfort, but only BMP was associated with stress levels. In sum, low BMP was associated with increased pain and stress levels in the majority of patients, and stress moderated the relationship between BMP and pain at the group level [22].

Extreme exertion, static muscle strain, and mental strain are also associated with stress responses where the muscles are kept contracted to an excessive extent [1]. Another such stress response is the contraction of muscles in cold temperatures to keep warm. All changes in fluid pressure inside an enclosed compartment can regulate the internal temperature. The heating modalities can potentially cause edema due to vasodilatation and locally increased fluid flow [23]. IFMFP will dynamically be affected by both internal temperature and extramuscular pressure (BMP). If BMP decreases (low pressure), the difference across the fasciomembrane against IFMFP increases, and vice versa. This alteration of tissue swelling can cause inhibited circulation and ischemic pain and can stimulate pain; this might be a possible mechanism for the perceived weather-related pain. The correlation between both increased or decreased BMP versus the degree of IFMFP over the fasciomembrane may explain the differences found at the individual level

## Conclusions

The aim of this review has been to establish a novel theoretical approach to the extended clinical manifestations of myalgias such as TrP, CECS, OTS, and DOMS. This new model also provides an explanation of the influence in both affected and associated tissue. This review builds on and suggests that intrafasciomembranal conditions related to the degree of fluid pressure (IFMFP) are important for the depth and length of the myofascial tissue. The increased IFMFP produces a swelling and shortens the affected tissue at all levels (muscle fibers, fiber bundles, skeletal muscles, and compartments). Both affected (local Influence) and associated tissue (distal influence) in myofascial chains can potentially be exposed to forces that mechanically affect nerve endings (nociceptors) and give reduced circulatory conditions with ischemic pain and reduced metabolism, leading to pain and loss of tissue function. The mechanical stress on the myofascial tissue provides a valid explanation for the thickening of the fascia and densification, which together with an increased IFMFP can also change the lines of force through the body and explain referred pain. This model, which takes into account IFMFP, can also provide a valid theory that internal fluid pressure and external BMP and temperature affect perceived weather-related pain.

In the context of the patient's dysfunction of free movement and perceived pain, this new approach could potentially change the view of many myalgias, dysfunctions, and their origins in the human body. In an even broader perspective and through the concepts of biotensegrity and fascintegrity, the possible impact may be even greater than discussed in this article. No conclusive research has been found on whether an increased IFMFP in muscle tissue independently shortens the tissue length. Further research is recommended.
